# The discovery of a novel compound with potent antitumor activity: virtual screening, synthesis, biological evaluation and preliminary mechanism study

**DOI:** 10.18632/oncotarget.15601

**Published:** 2017-02-21

**Authors:** Yuanyuan Jin, Linhu Li, Zhaoyong Yang, Mingliang Liu, Huiyuan Guo, Weiyi Shen

**Affiliations:** ^1^ Institute of Medicinal Biotechnology, Chinese Academy of Medical Sciences and Peking Union Medical College, Beijing 100050, China; ^2^ Zhejiang Starry Pharmaceutical Co. Ltd., Xianju 317300, China

**Keywords:** anti-tumor, synthesis, virtual screening, farnesyltransferase inhibitor

## Abstract

Farnesyltransferase has been regarded as a promising drug target against cancer as it is critical for membrane association of several signal transduction proteins. In this study, a novel farnesyltransferase inhibitor (IMB-1406) was identified through virtual screening. It exhibits stronger potency (IC_50_s: 6.92–8.99 μM) than Sunitinib against all of the tested cancer cell lines. Preliminary studies on mechanism reveal that IMB-1406 induces apoptosis in HepG2 cells by arresting the cell cycle at the S phase, altering anti- and pro-apoptotic proteins leading to mitochondrial dysfunction and activation of caspase-3. This anti-tumor effect is most probably related to the inhibition of farnesyltransferase as indicated by molecular docking. Overall, IMB-1406 is a novel lead compound with potent antitumor activity and deserves further structural modifications.

## INTRODUCTION

Cancers still remain the second leading cause of death worldwide after cardiovascular diseases [1]. Many classes of antitumor agents, especially tyrosine kinase (TK) inhibitors, have been introduced to the market during the last decade, and more candidates are now under development [2]. However, given the emergence of drug resistance and the low success rate in clinical development, the discovery of novel chemical compounds with a completely new mode of action is clearly needed.

The *Ras* genes encode more than 100 proteins, collectively known as the Ras superfamily, which are involved in the regulation of proliferation, differentiation, cell adhesion and apoptosis of cells. Some of these proteins are small G proteins that transducer signals from cell-surface receptors, such as growth factor receptors, and then transmit the signals to several different pathways, ultimately affect mitogenic functions such as DNA synthesis, cytoskeletal organization and lipid metabolism. Disruption of these signal transduction pathways through mutation of the *Ras* genes involved in many tumor types [3].

Farnesylation is a type of lipid modification called protein prenylation that is critical for biological functionality, including membrane association of several signal transduction proteins. Oncogenically mutant forms of human Ras superfamily proteins are associated with 30% of all human cancers; the transforming ability of these mutants is dependent upon farnesylation. More recently, farnesyltransferase has become popular drug targets [4, 5]. Inhibitors of farnesyltransferase cause tumor regression in animals and are currently being evaluated in clinical trials for the treatment of human cancers [6–8]. Farnesyltransferase inhibitors (FTIs) that have been or are being investigated in clinical trials include lonafarnib (SCH66336; Sarasar^TM^; Schering-Plough), tipifarnib (R115777; Zarnestra^TM^; Ortho BiotechProducts), L778123 (Merck), BMS-214662 (Bristol-MyersSquibb), and salirasib (S-*trans,trans*-farnesylthiosalycilic acid, FTS, Concordia Pharmaceuticals) [9]. However, none of these have yet been approved by FDA for clinical treatments. Therefore, it is imperative to develop more new FTIs.

Computer-aided drug design is one of most effective methods in developing new drugs. Many reports have indicated that computational method is a time-saving and effort-saving approach to discover new candidates including molecular docking [10], virtual screening [11], and 3DQSAR [12, 13]. Virtual screening (VS) has emerged as a powerful approach to complement the array of existing high-throughput screening technologies [14, 15]. Using computer-aided VS, potential leads can be rapidly identified in silico in order to narrow the range of molecules to be tested *in vitro* and *in vivo*. In this study, virtual screening was used as a 3D query in an in-house chemical database over 900 compounds screening to retrieve potential FTIs.

## RESULTS AND DISCUSSION

### Virtual screening

**Figure 1:**
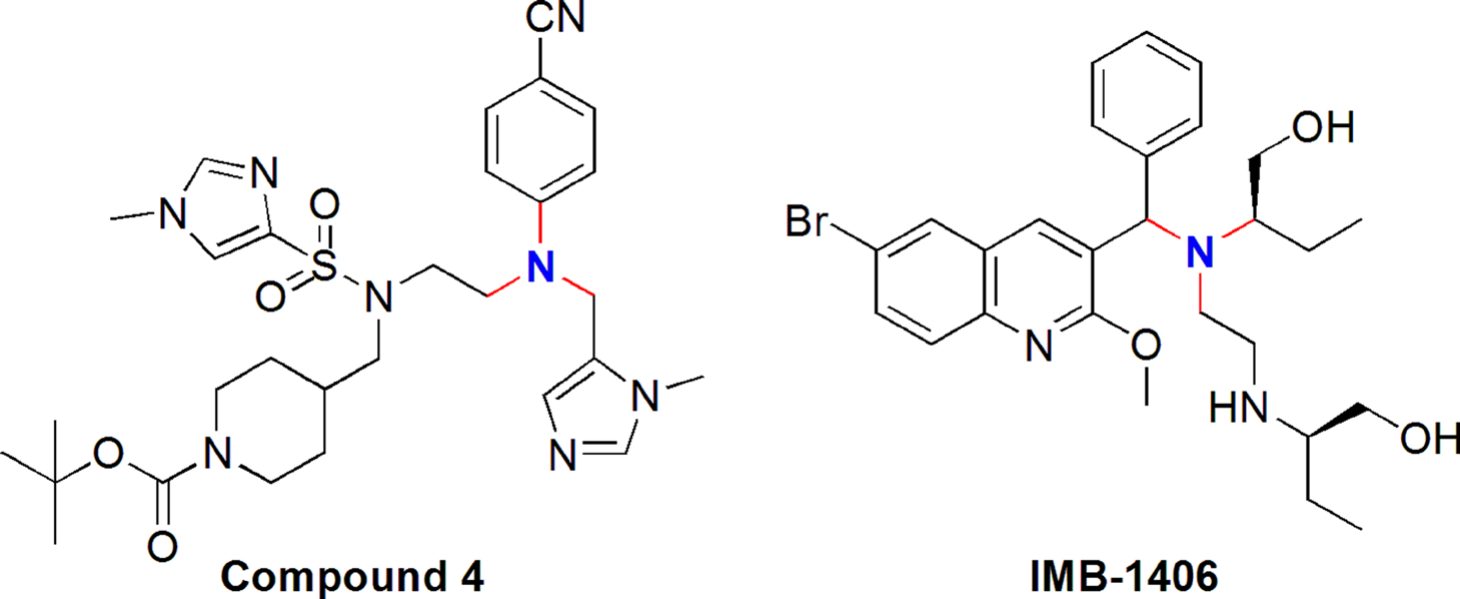
Chemical structures of Compound 4 and the most active compound IMB-1406 used in the present study

Virtual screening of chemical databases can serve the purpose of finding novel potential leads suitable for further development. Molecular docking technology which has been increasingly used in the course of drug research and development is the representative of receptor-based virtual screening [16, 17]. In this paper, molecular docking experiments were carried out using Discovery Studio 2.5 software (Accelrys Inc., San Diego, CA, USA) with fully automated docking tool using “CDOCKER” protocol [18–22] employing CHARMm force field to investigate the possible inhibitors for farnesyltransferase. The CDOCKER energy (-(protein-ligand interaction energies)) of best poses docked into the receptor of all the 900 compounds was calculated and compared with that of Compound 4 which is a new potent farnesyltransferase inhibitor based on the ethylenediamine scaffold. As a result, IMB-1406 was found to have the highest CDOCKER energy and similar structural framework to the crystallized inhibitor (Compound 4) [23] of farnesyltransferase, as shown in Figure 1.

**Figure 2:**
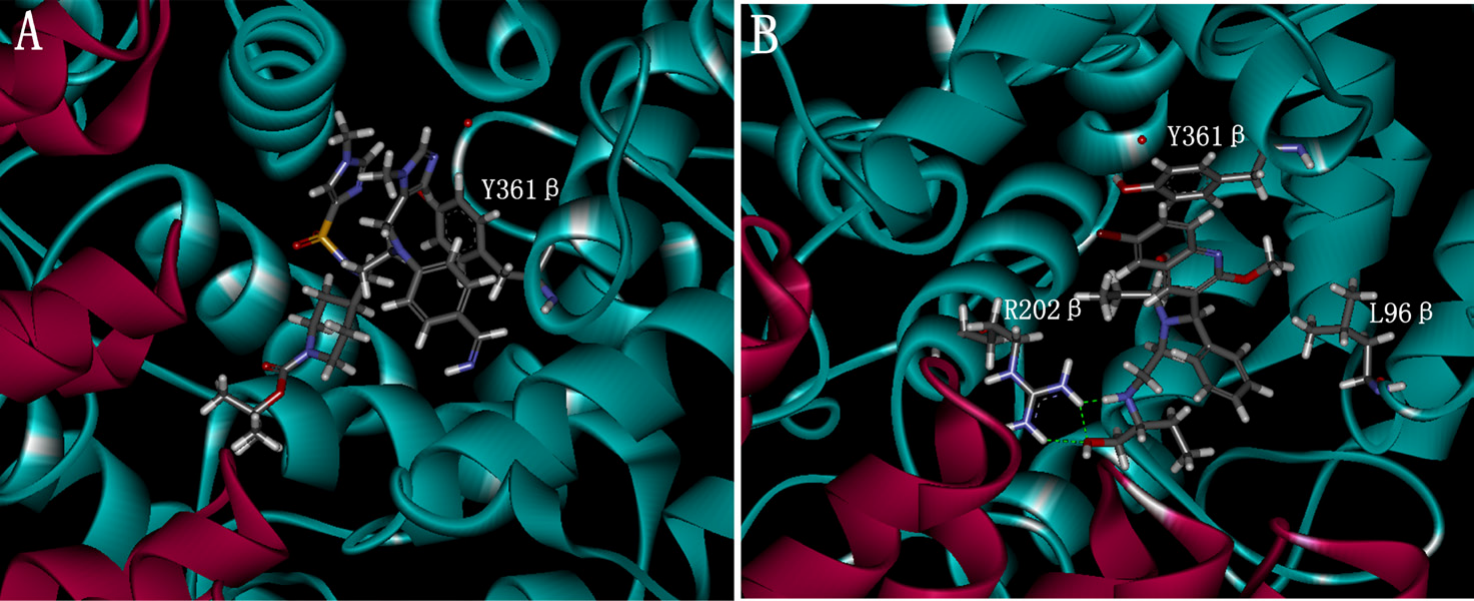
Docking of the Compound 4 (**A**) and the compound IMB-1406 (**B**) with farnesyltransferase.

According to the docking result, the most active compound IMB-1406 and the Compound 4 are found to bind in the active pocket of farnesyltransferase receptor in a similar conformation (Figure 2). As described above, both the para-benzonitrile moiety of the Compound 4 and the 6-bromo-2-methoxyquinoline one of IMB-1406 are oriented toward the product exit groove and are partially stabilized by a stacking interaction with Y361β. In Figure 2B, there are three hydrogen bonding interactions between the N22 and O33 positions of IMB-1406 and R202β. This is consistent with the -CDOCKER_INTERACTION_ENERGY between the two compounds and the farnesyltransferase. The highest -CDOCKER_INTERACTION_ENERGY was 52.24 for IMB-1406 and that of Compound 4 was 57.22, suggesting that IMB-1406 could have similar affinity with the Compound 4 and the farnesyltransferase might be one of the possible targets for IMB-1406.

### Chemical synthesis

**Scheme 1:**
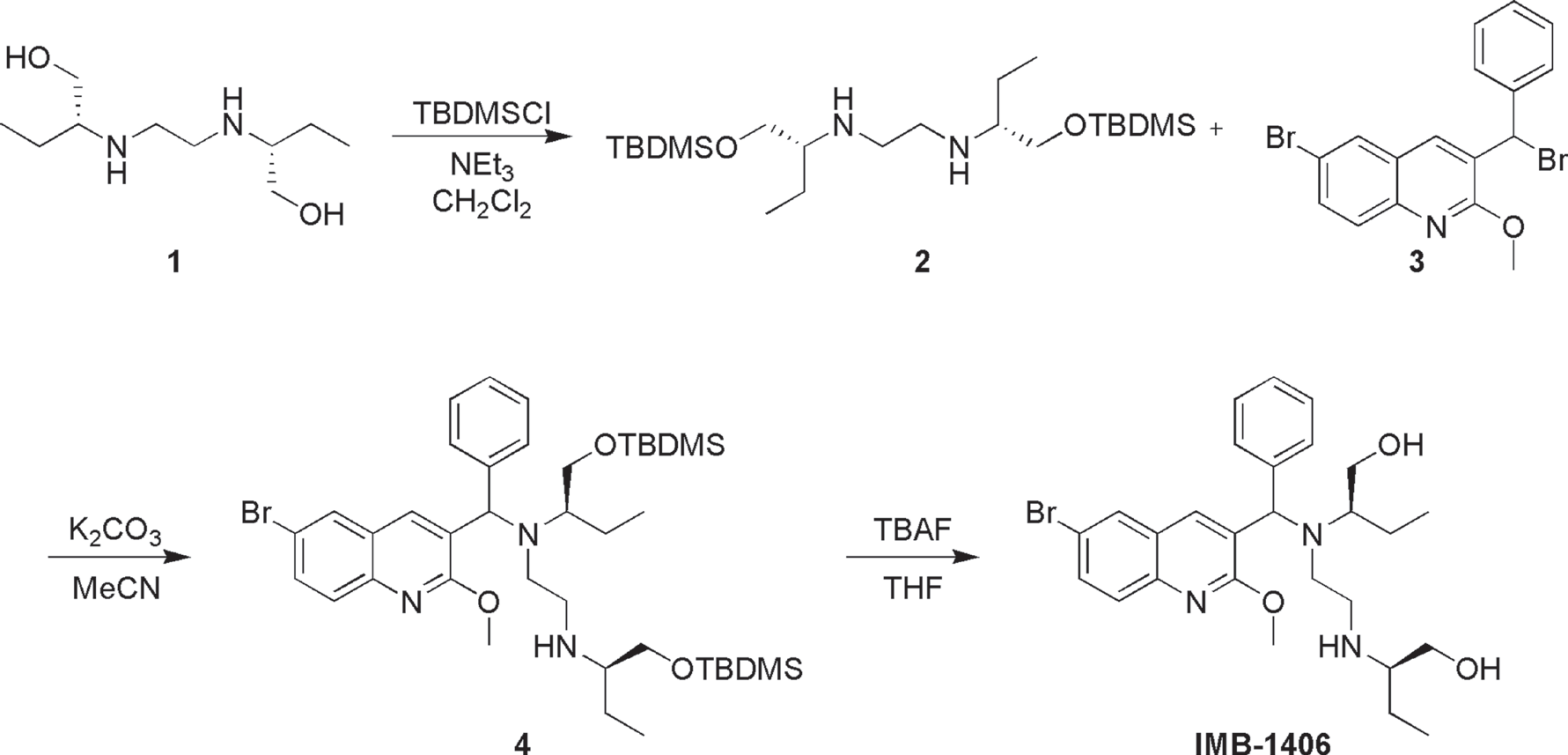
Synthesis of target compound IMB-1406 (2R)-2-(2-(((6-bromo-2-methoxyquinolin-3-yl)(phenyl)methyl)((R)-1-hydroxybutan-2-yl)amino)ethylamino)butan-1-ol (IMB-1406); ^1^H NMR (400 MHz, DMSO-*d6*) δ 8.70 (s, 1H), 8.12 (d, *J* = 2.0 Hz, 1H), 7.73–7.66 (m, 2H), 7.34 (d, *J* = 7.2 Hz, 2H), 5.49 (s, 1H), 4.37 (brs, 1H), 3.89 (s, 3H), 3.50–3.09 (m, 5H), 2.75-2.60 (m, 3H), 2.33-2.22 (m, 2H), 2.09–2.06 (m, 1H), 1.33–1.09 (m, 4H), 0.74 (t, *J* = 7.2 Hz, 3H), 0.65 (t, *J* = 7.2 Hz, 3H); ^13^C NMR (101 MHz, DMSO-*d6*) δ 160.13, 143.42, 141.47, 136.28, 131.91, 129.69, 129.56, 129.05, 128.44, 128.03, 126.95, 126.49, 116.37, 62.41, 62.34, 60.24, 53.59, 47.08, 23.47, 21.29, 11.93, 9.82. HRMS (m/z) (ESI): calcd for C_27_H_37_BrN_3_O_3_ [M+H] ^+^: 530.20183; found: 530.20307.

Detailed synthetic pathway to compound IMB-1406 is depicted in Scheme 1. Treatment of ethambutol (EMB, 1) with tert-butyldimethylsilyl chloride (TBDMSCl) gave the desired bis-TBDMS-protected compound 2. The coupling of the resulting diamine 2 with intermediate 3 (22) and then deprotection of the hydroxyl groups with tetrabutylammonium fluoride (TBAF) in tetrahydrofuran (THF) yielded the target compound IMB-1406 (Scheme 1).

### Antitumor activity

For preliminary screening of antitumor activity, IMB-1406 was first investigated for cytotoxic activity against A549 (lung adenocarcinoma), MCF-7 (breast cancer), DU145 (prostate carcinoma) and HepG2 (liver carcinoma). Then the compound was further evaluated for its *in vitro* anti-tumor activity against the aforementioned four human cancer cell lines by the MTT assay. The inhibition rate and IC_50_ values were compared with those of Sunitinib, a multi-targeted receptor TK inhibitor [24] (Table 1). IMB-1406 exhibits remarkable anti-tumor activity (inhibition rate: 99.93%–100.39%, IC_50_s: 6.92–8.99 μM) which is better than Sunitinib (inhibition rate: 86.51%–100.02%, IC_50_s: 7.60–10.36 μM) against these cancer cell lines, and thus is selected as the lead compound for preliminary mechanism study on the growth inhibition of HepG2 cells.

**Table 1:** *In vitro* anti-tumor activity of IMB-1406 and sunitinib against four cell lines

Cell lines	Inhibition rate (30 μM)		IC_50_ (μM)	
	IMB-1406	Sunitinib	IMB-1406	Sunitinib
**A549**	100.07%	100.02%	8.99	10.36
**HepG2**	99.98%	98.61%	6.92	7.60
**DU145**	99.93%	86.51%	7.89	7.99
**MCF7**	100.39%	88.89%	8.26	8.65

### Effects of IMB-1406 on cell cycle of HepG2 cells

**Figure 3:**
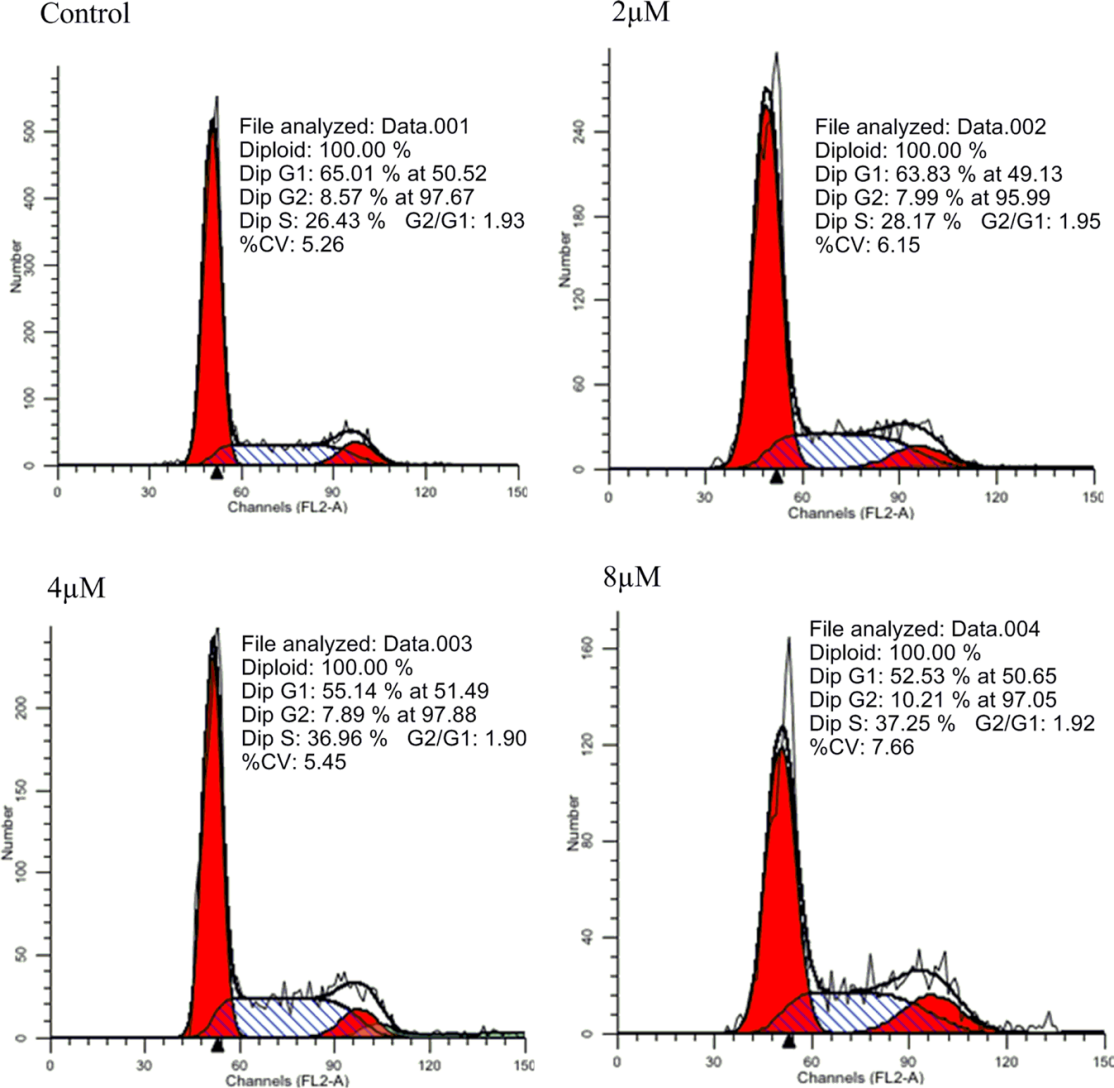
Effects of IMB-1406 on cell cycle of HepG2 cells HepG2 cells were treated with the indicated concentrations of IMB-1406 for 72 h and stained with PI, followed by flow cytometry analysis.

To understand the mechanisms underlying the action of IMB-1406, the effects of IMB-1406 on HepG2 cell cycle were examined. HepG2 cells were treated with the indicated concentrations of IMB-1406 for 72 h and stained with propidium iodide (PI), followed by flow cytometry analysis. As shown in Figure 3, the percentages of HepG2 cells in the S phase are 28.17% (2 μM), 36.96% (4 μM) and 37.25% (8 μM), respectively, which are significantly higher than that of the vehicle treated controls (26.43%). Hence, IMB-1406 inhibits the growth of the cancer cells by inhibiting the cell cycle via S-phase arrest.

### Effects of IMB-1406 on induction of HepG2 cell apoptosis

**Figure 4:**
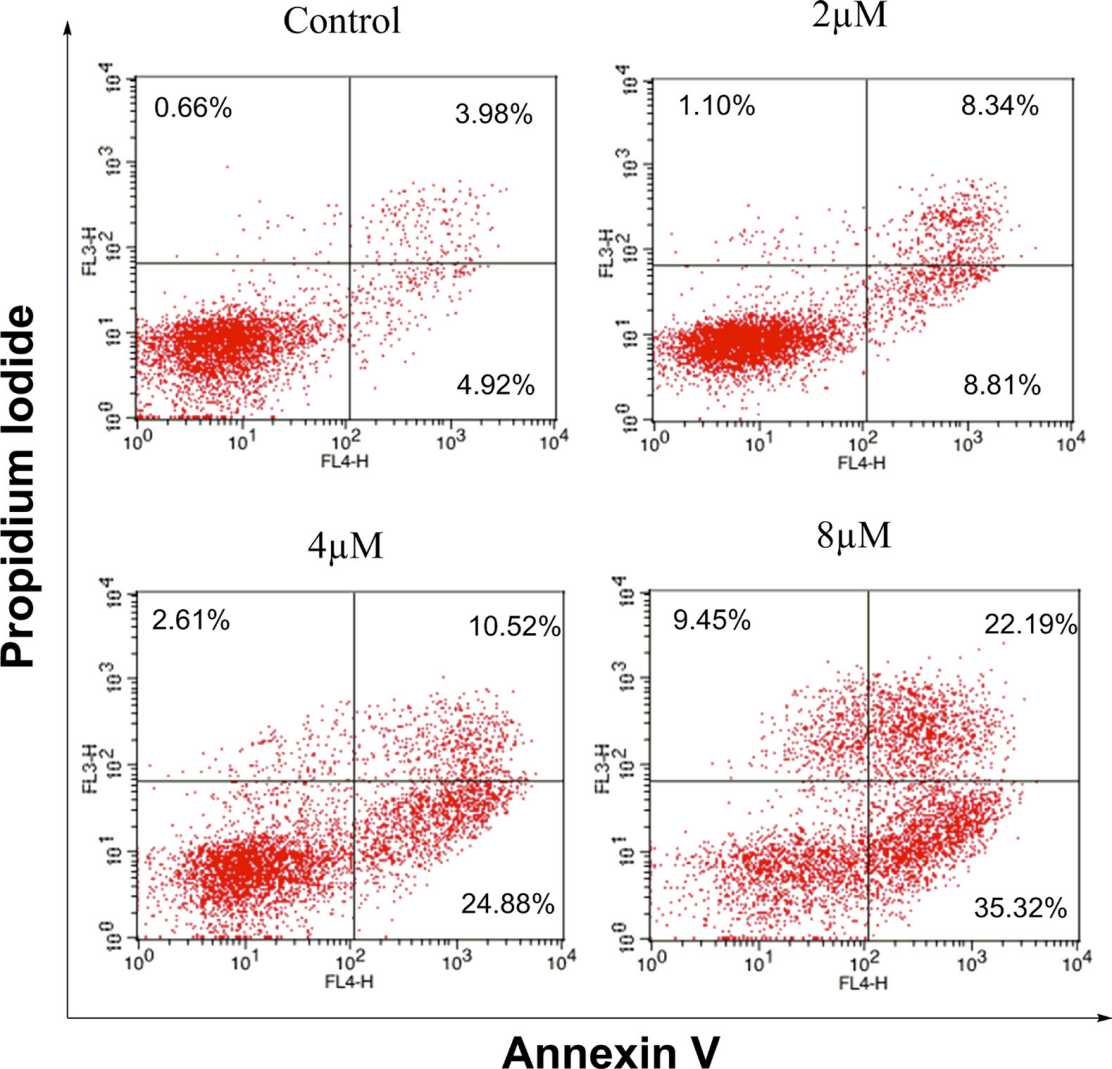
Effects of IMB-1406 on induction of HepG2 cell apoptosis HepG2 cells were treated with the indicated concentrations of IMB-1406 for 72 h and stained with Annexin V/PI, followed by flow cytometry analysis. The percentage of cell positive for PI and/or Annexin V-FITC are reported inside the quadrants.

Apoptosis plays a central role in cancer treatment, since its induction in cancer cells is critical to a successful therapy [25, 26]. It is thus believed that apoptosis assay may provide important information to the preliminary investigation of the mode of action. The impact of IMB-1406 on the apoptosis of HepG2 cells was examined by Annexin V/PI staining followed by flow cytometry analysis. As shown in Figure 4, treatment of HepG2 cells with IMB-1406 for 72 h results in an increase of the apoptosis ratios from 8.9% (untreated vehicle control) to 17.15% (2 μM), 35.40% (4 μM) or 57.51% (8 μM), indicating that IMB-1406 is able to induce apoptotic cell death in HepG2 cells in a concentration-dependent manner.

### Effects of IMB-1406 on mitochondrial membrane potential of HepG2 cells

**Figure 5:**
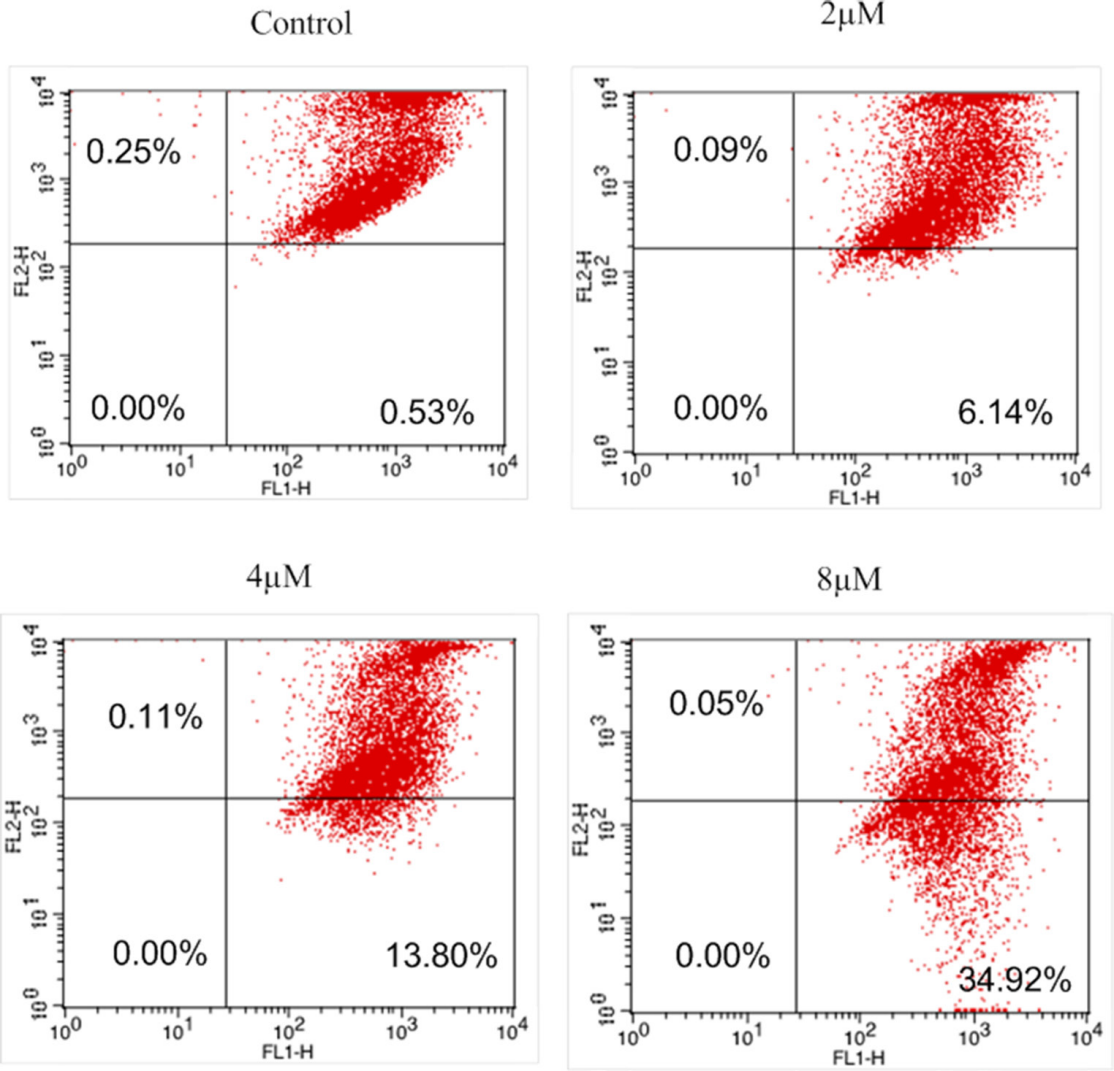
JC-1 mitochondrial membrane potential staining of IMB-1406 in of HepG2 cells HepG2 cells were treated with the indicated concentrations of IMB-1406 for 72 h and stained with JC-1, followed by flow cytometry analysis.

In order to determine the plausible pathway by which IMB-1406 triggers cell apoptosis, the changes of mitochondrial membrane potential were assessed. The uptake of 5,5′,6,6′-tetrachloro -1,1′,3,3′- tetraethylbenzimidazol-carbocyanine iodide (JC-1), a lipophilic cationic fluorescent dye accumulated in mitochondria, is positively correlated with the mitochondrial membrane potential [27]. HepG2 cells were treated with different concentrations (0, 2, 4, 8 μM) of IMB-1406 for 72 h prior to staining with JC-1. Then the numbers of cells with collapsed mitochondrial membrane potential in different cell groups were determined by flow cytometric analysis. As shown in Figure 5, the percentage of cells with typical apoptotic morphology increases in a dose-dependent fashion (0.53%, 6.14%, 13.8% and 34.92%, respectively). Our results reveal that incubation with IMB-1406 increases the number of cells with collapsed mitochondrial membrane potentials and induces apoptosis.

### Effects of IMB-1406 on the levels of Bax, Bcl-2 and caspase-3

**Figure 6:**
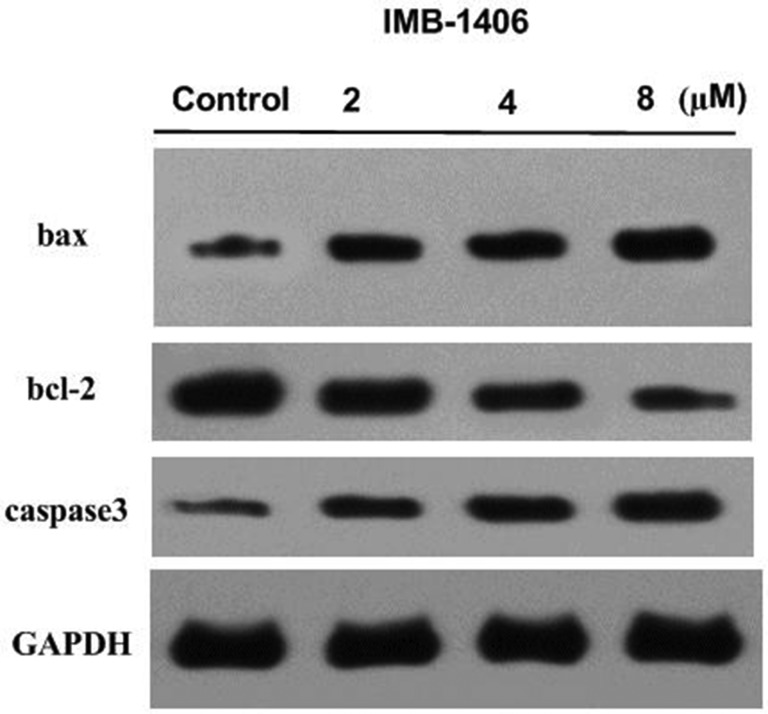
Effects of IMB-1406 on the levels of Bax, Bcl-2 and caspase-3 HepG2 cells were treated with IMB-1406 at 2 μM, 4 μM and 8 μM for 72 h. The cell lysates were collected and expression levels of Bax, Bcl-2 and caspase-3 were determined by western blot analysis. GADPH was used as internal control.

Mitochondria play an essential role in cell death signal transduction. The mitochondria-dependent apoptotic pathway is regulated by the Bcl-2 family of pro- and anti-apoptotic proteins, which induce the permeabilization of the mitochondrial outer membrane, resulting in the activation of the caspase cascade and the induction of apoptotic cell death [28, 29]. To explore the possible role of a mitochondrial-related pathway in IMB-1406-induced apoptosis, the effects of IMB-1406 on the levels of Bax, Bcl-2 and caspase-3 were examined by Western blot analysis. As shown in Figure 6, IMB-1406 treatment shows concentration-dependent effects in upregulating the expressions of Bax and downregulating Bcl-2. In addition, caspase-3 is activated in a concentration-dependent manner after treatment with IMB-1406 at 2 μM, 4 μM and 8 μM for 72 h. These results reveal that IMB-1406 induces apoptosis in HepG2 cancer cells via a mitochondria-dependent pathway.

## MATERIALS AND METHODS

### Virtual screening

The representative crystal structure of farnesyltransferase (PDB ID 3E30) was obtained from the Protein DataBank. Initially there was a pretreatment process for both the ligands and the enzymes. For ligand preparation, all the duplicate structures were removed and options for ionization change, tautomer generation, isomer generation, Lipinski filter and 3D generator were set true. For enzyme preparation, the whole enzyme was selected, waters were removed and hydrogen atoms were added to it. The pH of theprotein was set in the range of 6.5 to 8.5. Then we defined the enzyme as a total receptor and the binding site was defined by selecting the important amino acids for catalysis to generate a 10 Å radius sphere by the operation of Define and Edit Binding Site. Docking of compounds into enzymes with CDOCKER was done using the default parameters. The pose with the top -CDOCKER_INTERACTION_ENERGY was chosen for analyzing the binding features.

### Chemistry

All the reagents were purchased from commercial suppliers and were used without further purification unless otherwise indicated. Reaction courses were monitored by TLC on silica gel precoated F254 Merck plates. Developed plates were examined with UV lamps (254 nm). ^1^H-NMR and ^13^C-NMR spectra were taken on a Bruker spectrometer using TMS as the internal standard. DMSO-d_6_ and CDCl_3_ were used as the solvent. Mass spectra were measured on an Agilent 1100 series.

### General procedure for the synthesis of intermediate 2

To a solution of ethambutol dihydrochloride (1, 2.77g, 10 mmol) and Et_3_N (5.70 ml, 44 mmol) in anhydrous DCM (100ml) was added dropwise a solution of tert-butyldimthylsilyl chloride (3.30 g, 22 mmol) in anhydrous DCM (20 ml) at 0°C. After the addition, the mixture was stirred at room temperature for 2 h and then washed with water (150 ml), satd. NaHCO_3_ (100 ml) and brine (100 ml). The organic fractions were dried over anhydrous magnesium sulfate. The solvent was removed in vacuo to yield 2 (4.72 g, 80.5%) as a colourless oil.

### General procedure for the synthesis of intermediate 4

Anhydrous potassium carbonate (0.83 g, 3 mmol) was added to a solution of 2 (1.30 g, 3 mmol) and 3 (200 mg, 0.5 mmol) in acetonitrile (10ml). The resulting mixture was reflux for overnight, after which the reaction mixture was poured into water (20 ml), and extracted with DCM (3 × 20 ml). The combined organic extracts were washed with satd. NaHCO_3_ (2 × 30 ml) and brine (30 ml), dried over anhydrous magnesium sulfate and solvents removed in vacuo to obtain the crude reaction mixture. Further purification was performed by silica flash column chromatography eluting with DCM/MeOH (95:5), affording 4 as a light yellow oil (0.26 g, 69.5%).

### General procedure for the synthesis of IMB-1406

The solution of compound 4 (0.28 g, 0.37 mmol) in THF (5 ml) and 1M TBAF (in THF, 1.37 ml, 1.48 mmol) were allowed to react at room temperature for 10h, then the reaction mixture was concentrated and purified silica flash column chromatography (0–10% (v/v) MeOH in DCM). The collected fraction were evaporated to afford the alcohol IMB-1406 as a white solid (90.2 mg, 45.9%).

### Biological assay

### Cell lines and culture

Cell lines of A549, MCF-7, DU145 and HepG2 were provided by Nanjing KeyGen Biotech. Inc. All of these cells were cultured in Dulbecco's modified Eagle's medium (DMEM, Invitrogen) containing 10% calf serum (CS) and grown in a humidified atmosphere of 5% CO_2_ at 37°C.

### Reagents

The Annexin V- APC/7-AAD Apoptosis Detection kit, Cell cycle detection kit, Mitochondrial membrane potential detection kit, SDS-PAGE gel preparation kit and the enhanced chemiluminescence (ECL) kit were purchased from Nanjing KeyGen Biotech Co., Ltd. (Nanjing, China). DMSO and MTT were purchased from Sigma-Aldrich (Merck Millipore, Darmstadt, Germany). Other reagents were purchased from Beyotime Institute of Biotechnology (Jiangsu, China).

### MTT assay

Cell growth inhibition was determined using a colorimetric MTT assay. The assay was conducted in a 96-well plate with a cell density of 8 × 10^3^ cells per well with an incubation period of 24 h. The cells were then incubated with different concentrations of IMB-1406 (0, 20, 40, 80, 160 and 320 mg/l) in triplicate. After 24, 48 and 72 h of incubation, the cells were incubated with medium containing MTT for 4 h, and the formazan crystals were dissolved with 150 μl DMSO. The dark blue MTT crystals were dissolved by agitating the plates at room temperature for 10 min, and the absorbance was then measured at 490 nm with a microplate reader (OLYMPUS IX51, Japan).

### Apoptosis detection assay

HepG2 cells were seeded into 6-well plates and treated with IMB-1406 at different concentrations for 72 h. Apoptosis was then detected by the following procedures.

### Flow cytometry analysis of Annexin V-APC/7-AAD staining

HepG2 cells were inoculated in 6-well plates and then processed accordingly after they adhered. Cells were treated with IMB-1406, after 72 h the cells were collected by trypsin without EDTA, washed twice with PBS. The Annexin V-APC/7-AAD Apoptosis Detection kit (KGA1024, KeyGEN, Nanjing, China) was used for cell staining and flow cytometry (Becton-Dickinson FACS Calibur) following the manufacturer's instructions. The percentage of apoptotic cells was determined using FACS flow cytometry and associated software (BECKMAN). Experiments were repeated three times, and the results are displayed as histograms.

### Cell cycle detection

Cells in the logarithmic phase of growth were digested by trypsin and centrifuged. After counting, the cells were inoculated in 6-well plates. Cells were processed accordingly after they adhered. Whereafter, the cells were collected and washed with 1 × PBS, and then 70% ethyl alcohol was added for fixation overnight. After the supernatant was removed, the cells were washed with 1 × PBS. Once the RNA enzyme was added, the cells were placed at 37°C for 30 minutes. After digestion, PI was added for staining. Then, the cells were placed in the dark at 4°C for 30 minutes. The PI-stained protoplasts were measured by flow cytometry with an excitation wavelength of 488 nm.

### Measurement of mitochondrial membrane potential

HepG2 cells were inoculated in 6-well plates and then processed accordingly after they adhered. Cells were treated with IMB-1406, after 72 h the cells were collected, washed once with PBS and resuspended in fresh medium containing 5, 5′,6, 6′tetrachloro-1, 1′, 3, 3′-tetraethylbenzimidazol-carbocyanine iodide (JC-1). After incubation at 37°C for 15∼20 minutes under a humidified atmosphere containing 5% CO_2_, cells were analyzed by flow cytometry with an excitation wavelength of 488 nm.

### Detecting protein by western blot

Cell total protein extraction was performed, followed by sodium dodecyl sulfate–polyacrylamide gel electrophoresis, transmembrane antibody incubation, and color development.

## CONCLUSIONS

In summary, a new compound (IMB-1406) was identified, synthesized and characterized by ^1^HNMR, ^13^CNMR and MS. IMB-1406 was found to have considerable potency (IC_50_s: 6.92–8.99 μM) which is better than Sunitinib against all four cancer cell lines tested. Moreover, preliminary study on mechanism revealed that IMB-1406 induces apoptosis in HepG2 cells by arresting the cell cycle at the S phase, altering anti- and pro-apoptotic proteins leading to mitochondrial dysfunction and activation of caspase-3. Meanwhile, molecular docking study suggested one of the possible targets for IMB-1406 was protein farnesyltransferase. Together, IMB-1406 is a novel conjugate with potent antitumor activity, and it could contribute to a better understanding of structure-based drug design to facilitate drug discovery. Based on this, further structural modifications, SAR studies and enzyme inhibitory activity of IMB-1406 are currently in progress.
